# Delta-like 3 is silenced by methylation and induces apoptosis in human hepatocellular carcinoma

**DOI:** 10.3892/ijo.2013.1778

**Published:** 2013-01-17

**Authors:** KENTARO MAEMURA, HIROHIDE YOSHIKAWA, KAZUTAKE YOKOYAMA, TERUO UENO, HITOMI KUROSE, KAZUHISA UCHIYAMA, YOSHINORI OTSUKI

**Affiliations:** 1 Department of Anatomy and Cell Biology, Osaka Medical College, Takatsuki, Osaka;; 2 Department of Internal Medicine, Sassa General Hospital, Nishi-tokyo, Tokyo;; 3 Department of General and Gastroenterological Surgery, Osaka Medical College, Takatsuki, Osaka, Japan; 4 Central Research Unit, Osaka Medical College, Takatsuki, Osaka, Japan

**Keywords:** Delta-like 3, hepatocellular carcinoma, methylation, apoptosis

## Abstract

The genetic and epigenetic events of hepatocarcinogenesis are relatively poorly understood. By analyzing genes from human hepatocellular carcinoma (HCC) with restriction landmark genomic scanning, several aberrantly methylated genes, including *Delta-like 3* (*DLL3*), have been isolated. In this study, we investigated the function of DLL3 in hepatocarcinogenesis. Methylation of the *DLL3* gene in HCC cell lines was investigated with methylation-specific PCR and expression of DLL3 mRNA in HCC cells was examined by RT-PCR. Reactivation of DLL3 expression by treatment with a demethylating agent was examined in methylation-silenced HuH2 cells. Human DLL3 cDNA was cloned and DLL3 function was examined by restoring DLL3 expression in HuH2 cells. The effects of DLL3 on cell growth were evaluated by colony formation assay. Induction of cell death by overexpression of DLL3 was examined by flow cytometric assay using Annexin V and PI. Apoptotic cells were detected by TUNEL staining and the amount of single-stranded DNA was measured by ELISA. As a result, the promoter region of the *DLL3* gene was methylated in four of ten HCC cell lines. This aberrant methylation correlated well with the suppression of RNA expression and a demethylating agent reactivated DLL3 expression in methylation-silenced HCC cells. Interestingly, the restoration of DLL3 in the methylation-silenced HuH2 cells led to growth suppression on colony formation assay. Flow cytometric assay with Annexin V and PI showed that this growth suppression by DLL3 expression is associated with the induction of apoptosis. Furthermore, these apoptotic effects were confirmed by TUNEL staining and measurement of single-stranded DNA. These results suggest that DLL3 was silenced by methylation in human HCC and that it negatively regulates the growth of HCC cells.

## Introduction

Hepatocellular carcinoma (HCC) is one of the most common malignant tumors worldwide and most HCC arises from chronic liver disease, which is associated with liver cirrhosis. Etiological factors for hepatocarcinogenesis include chronic hepatitis B or hepatitis C virus infection, long-term exposure to aflatoxin B1 in food, alcohol addiction and hemochromatosis. The precise molecular mechanisms for its development beyond initiation have not been elucidated, but as for many other tumors, the development and progression of HCC is a multistep process and activation of oncogenes and inactivation of tumor suppressor genes caused by genetic or epigenetic aberrance are involved in carcinogenesis (1). Among these, epigenetic inactivation of tumor suppressor genes by hypermethylation of CpG islands in promoter sequences plays an important role (2–4). Yoshikawa *et al*(6) compared genomic *Not*I restriction fragments between normal and HCC tissues by restriction landmark genomic scanning (RLGS) analysis and isolated several aberrantly methylated genes, such as *suppressor of cytokine signaling-1* (*SOCS-1*), *SOCS-3* and *apoptotic speck protein-like* (*ASCL*) (5–10).

Here, we demonstrate that the *Delta-like 3* (*DLL3*) gene was aberrantly methylated in its CpG island in HCC cells. DLL3 is a member of Delta/Serrate/Lag2 (DSL) ligands for Notch receptors and plays a role in Notch signaling. Five DSL ligands (DLL1, 3, 4; and Jagged 1 and 2) and four Notch receptors have been identified in mammals (11). Notch signaling is an evolutionarily conserved signaling pathway essential for embryonic development and regulates cellular processes such as differentiation, proliferation, survival and apoptosis in various cell types (12,13). However, DLL3 is the most structurally divergent DSL ligand and function of DLL3 in Notch signaling is complicated by conflicting reports (14–16). DLL3 is expressed throughout the presomitic mesoderm and is localized to the rostral somatic compartments. Homozygous disruptions of Notch1 and DLL3 result in severe abnormalities in somitogenesis (17–19) and mutations in the human DLL3 homolog cause recessive skeletal abnormalities in spondylocostal dysostosis (20–22). The role of DLL3 in carcinogenesis has not been reported.

In this study, we sought to examine the silencing of DLL3 by methylation and to characterize its roles in HCC. Our data indicate that DLL3 is silenced by methylation and DLL3 expression is associated with cell growth suppression in HCC. Our findings confirm a novel function of DLL3 in hepatocarcinogenesis.

## Materials and methods

### Cell lines

Human HCC cell lines HuH1, HuH4 and HuH7 were purchased from the Japanese Culture Collection. HuH2 and Kim1 were gifts from the Department of Pathology, The Cancer Institute and the Japanese Foundation for Cancer Research. Hep3B and Li-7 were obtained from the cell resource center for Biomedical Research Institute of Development, Aging and Cancer, Tohoku University. FLC4 was a gift from Dr Seishi Nagamori. Cells were maintained in RPMI-1640 medium (Sigma-Aldrich, St. Louis, MO, USA) containing 10% fetal bovine serum (Invitrogen, Carlsbad, CA, USA) at 37°C under a 5% CO_2_ atmosphere. For DLL3-reactivation study, cells were treated with 1 μM 5-Aza-2′-deoxycytidine (5-Aza-dC) alone for 4 days or with 1 μM 5-Aza-dC for 4 days and 300 nM trichostatin A (TSA) for 1 day.

### Reverse transcription-PCR analysis

Total RNA of HCC cell lines was isolated using an RNeasy mini kit (Qiagen, Hilden, Germany), and cDNA was synthesized using the Superscript Preamplification System (Invitrogen). An aliquot of cDNA was subjected to amplification using *Taq* polymerase (Takara, Shiga, Japan). The primer sequences were CGAGCTGCAGAT CCACTCT and CGCCTCACATTCGTCCTC. The reaction was carried out for 35 cycles of denaturation at 94°C for 40 sec, annealing at 62°C for 40 sec and extension 72°C for 180 sec. An aliquot of PCR product was analyzed by 1.5% agarose gel electrophoresis, followed by ethidium bromide staining.

### Methylation-specific PCR (MSP)

Bisulfite modification of genomic DNA was performed as described previously (23). The methylation-specific primer sequences for *DLL3* were CGGGATTATTTACGTATGATTTC [nucleotides (nt) 103,584-103,606 in AC011500] and CCGACCCCAAAAA ACCAAAAACG (nt 103,686-103,708). The unmethylation-specific primer sequences were TGTGGGATTATTTA TGTATGATTTT (nt 103,582-103,606) and CCCAACCCCA AAAAACCAAAAACA (nt 103,686-103,709). An aliquot of bisulfite-modified DNA was amplified by PCR. PCR was carried out with preheating at 94°C for 120 sec and 80°C for 30 sec, followed by 30 cycles of 94°C for 40 sec, 60°C for 40 sec and 72°C for 60 sec. An aliquot of PCR product was analyzed by 4.0% agarose gel electrophoresis.

### Construction of expression vector

A full-length DLL3 cDNA was isolated from human liver RNA (BD Sciences, Rockville, MD, USA) by PCR and the product was cloned into the *Eco*RI site of a pcDNA 3.1/HisB vector (Invitrogen). A clone, designated pcDNA3-DLL3, showed an in-frame ligation and correct sequence.

### Colony formation assay

Cells (5.0×10^4^ for HuH2, 1.0×10^5^ for Kim1) were transfected with 4 μg of either pcDNA-DLL3 or pcDNA3.1 backbone vector. Colonies were selected in the presence of G418 (1,000 μg/ml for HuH2, 300 μg/ml for Kim1) for 4 weeks and colonies were photographed after staining.

### Flow cytometry analysis of cell death

HuH2 cells were transfected with either pcDNA-DLL3 or pcDNA3.1 backbone vector. After 48 h, cell apoptosis was analyzed with an Annexin V-FITC kit (Bender MedSystems, Burlingame, CA, USA) along with PI according to the manufacturer’s protocol. Briefly, both adhered and floating cells (5×10^5^/ml) were resuspended in binding buffer and incubated with Annexin V-FITC for 10 min at room temperature. Cells were then washed with PBS and incubated with PI (final concentration 1 μg/ml) solution and DNA contents of the cells were measured with a flow cytometer (Beckman Coulter, Brea, CA, USA).

### TUNEL analysis

TUNEL assay was performed using a kit (Roche Biochemicals Inc., Mannheim, Germany) according to manufacturer’s protocol. Briefly, cells were plated on 18×18 mm coverslips placed in a 6-well plate and transfected with 1 μg of either pcDNA-DLL3 or pcDNA3.1 backbone vector. After 48 h, cells were fixed with 4% paraformaldehyde, and permeabilized with 0.1% Triton X-100 after blocking endogenous peroxidase. TUNEL reaction mixture was added to the cells and cells were incubated with converter-POD before adding substrate solution. Over 3,000 cells were counted from 15 randomly selected fields under a microscope.

### Measurement of single-stranded DNA

DNA in apoptotic cells is sensitive to formamide and denatured DNA was detected with a monoclonal antibody against single-stranded DNA with an ApoStrand™ ELISA apoptosis detection kit (Enzo Life Sciences, Plymouth Meeting, PA, USA) according to the manufacturer’s protocol. Briefly, 3.0×10^3^ or 4.0×10^3^ cells were plated in a 96-well microplate and transfected with 50 ng of either pcDNA-DLL3 or pcDNA3.1 backbone vector. After 48 h, cells were fixed and dried to attach cells to the plate surface. Cells were then treated with formamide and heated at 56°C for 30 min and were then incubated with antibody mixture for 30 min after blocking non-specific binding sites. After washing, peroxidase substrate was added to each well and absorbance was measured at 405 nm with an ELISA reader (Corona Electric Co. Ltd., Ibaragi, Japan).

### Western blot analysis

Expression of cleaved Notch I was detected by western blot analysis. Cells were seeded onto 10-cm dishes and transfected with 4 μg of either pcDNA-DLL3 or pcDNA3.1 backbone vector. After 48 h, cells were solubilized in lysis buffer [20 mM Tris-HCl pH 8.0, 150 mM NaCl, 1% NP-40, 0.5% deoxycholic acid, 0.1% sodium dodecyl sulfate containing complete protease inhibitor cocktail (Roche Diagnostic GmbH, Mannheim, Germany)], followed by centrifugation at 14,000 rpm for 15 min at 4°C. Supernatants (20 μg) were resolved by electrophoresis and were transferred to Immobilon-P membrane (Millipore, Billerica, MA, USA). Cleaved Notch was detected by probing membrane with antibody against cleaved Notch (Cell Signaling Technology, Beverly, MA, USA). Horseradish peroxidase-labeled anti-rabbit IgG was used as a secondary antibody and chemiluminescent reaction was carried out using ECL plus western blotting detection reagents (GE Healthcare UK, Buckinghamshire, UK). Signals were detected with a LAS-3000 lumino image analyzer (Fuji Photo Film, Tokyo, Japan).

## Results

### mRNA expression and methylation status of DLL3 in HCC cells

**Figure 1:**
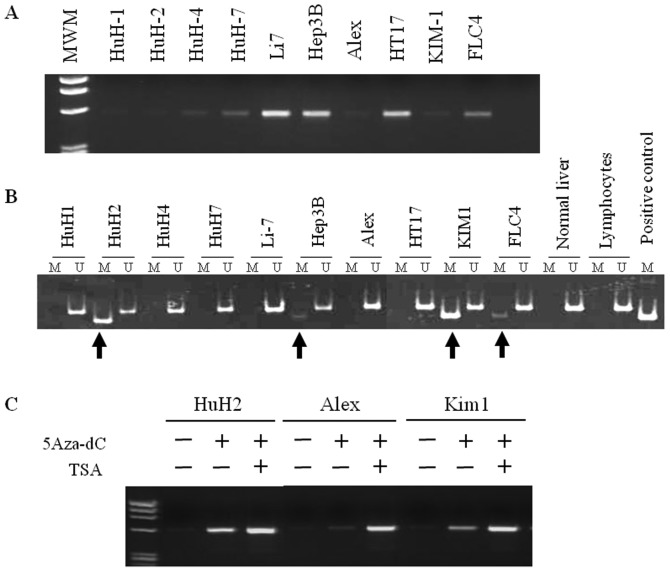
DLL3 mRNA expression and aberrant methylation of *DLL3* gene. (A) RT-PCR analysis of DLL3 mRNA from 10 HCC cell lines. No mRNA was detected in HuH1, HuH2, HiuH4, Alex or Kim-1 cells. (B) Methylation of *DLL3* was analyzed with MSP using the primer set around the translation start site in 10 HCC cell lines, a non-tumorous liver and lymphocytes. Visible bands in M-lanes (arrows) are methylated PCR products with methylation-specific primers. Visible bands in U-lanes are unmethylated PCR products with unmethylation-specific primers. (C) Three methylated cell lines (HuH2, Alex and Kim1) were treated with or without 5-Aza-2′-deoxycytidine (5-Aza-dC) and DLL3 mRNA expression was analyzed by RT-PCR.

We first analyzed DLL3 mRNA expression in 10 HCC cell lines. As shown in Fig. 1A, an amplified band was clearly detected in 6 cell lines (HuH4, HuH7, Li7, Hep3B, HT17 and FLC4) and a faint band was detected in Alex and Kim1 cells. No mRNA expression was observed in HuH1, HuH2 cells in addition to normal liver.

Methylation status of the *DLL3* CpG islands was then analyzed by MSP. A primer set was designed in exon1, which lies within the *DLL3* CpG islands. As shown in Fig. 1B, apparent methylation of *DLL3* was detected in four (HuH2, Hep3B, Kim1 and FLC4) cell lines among 10 cell lines tested with RT-PCR. Aberrant methylation of *DLL3* was not detected in normal liver tissue or lymphocytes.

We next analyzed whether a demethylating agent, 5-Aza-2′-deoxycytidine (5-Aza-dC), and a histone deacetylase inhibitor, trichostatin A (TSA), can reactivate DLL3 expression in HuH1, HuH2, HuH4, Alex and Kim1 cells. As shown in Fig. 1C, DLL3 expression was reactivated by 5-Aza-dC treatment in all cell lines tested. Although no methylation was detected in HuH1 and Alex cells with MSP, a clear amplified band was detected in these cells after treatment of 5-Aza-dC. Moreover, a robust effect was obtained by additional treatment with TSA in HuH2, Alex and Kim1 cells. These results suggest that expression of DLL3 is frequently suppressed or silenced in association with DNA methylation in HCC cells.

### Growth suppression by DLL3 restoration

**Figure 2:**
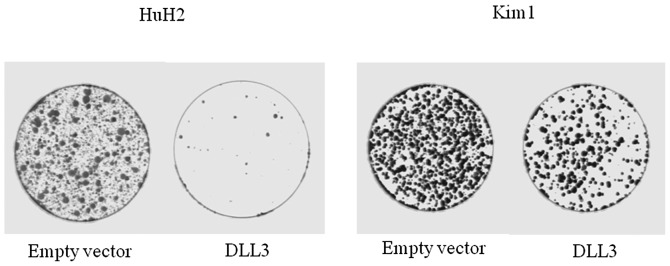
Growth suppression by DLL3. Methylation-silenced cells (HuH2 and Kim1) were transfected with either DLL3 expression vector or backbone vector and selected for 4 weeks with G418.

Colony formation assay was performed in order to investigate the effects of DLL3 overexpression on cell growth. As shown in Fig. 2, overexpression of DLL3 markedly suppressed colony formation in both HuH2 and Kim1 cells, in which DLL3 was silenced in association with DNA methylation. This suggests that DLL3 has cell growth activity in HCC cells.

### Induction of cell death by DLL3 expression

**Figure 3:**
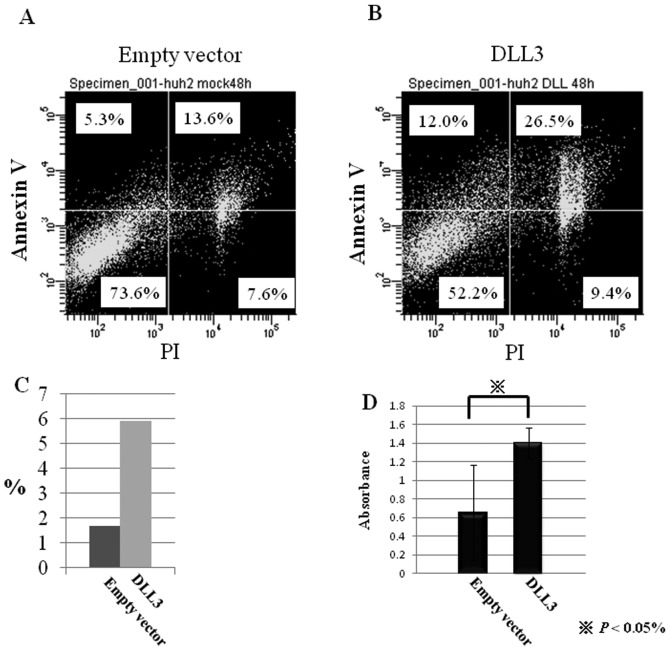
Induction of cell death by DLL3. Methylation-silenced HuH2 cells were transiently transfected with either (A) backbone vector or (B) DLL3 expression vector and cell death was detected by flow cytometry using PI and Annexin V staining. (C) Apoptotic cells were detected with the TUNEL method in HuH2 cells transfected with either backbone vector or DLL3 expression vector and numbers of TUNEL-positive cells were counted under microscopy. (D) Amount of single-stranded DNA was compared between control and DLL3-transfected cells by ELISA.

Flow cytometric analysis was performed in order to investigate the effects of DLL3 overexpression on cell death. Of the cells transfected with backbone vector, 21.2 and 18.9% were positive for PI and Annexin V, respectively (Fig. 3A and B). On the other hand, 35.9 and 38.5% of DLL3-transfected cells were positive for PI and Annexin V, respectively. These results suggest that overexpression of DLL3 induced cell death in HuH2 cells chiefly via apoptosis.

### Induction of apoptosis by DLL3 expression

In order to confirm the apoptotic effects of DLL3 on HuH2 cells, apoptotic cells were detected by the TUNEL method. As shown in Fig. 3C, the number of TUNEL-positive cells increased by transfection of DLL3. The percentage of TUNEL-positive cells in mock-transfected cells and DLL3-transfected cells was 1.68 and 5.93%, respectively. Furthermore, the amount of single-stranded DNA was significantly increased by transfection of DLL3 in HuH2 cells (Fig. 3D). These data support the notion that restoration of DLL3 induces apoptosis in HuH2 cells in which DLL3 is silenced by DNA methylation.

### Effects of DLL3 on Notch1 activation

**Figure 4:**
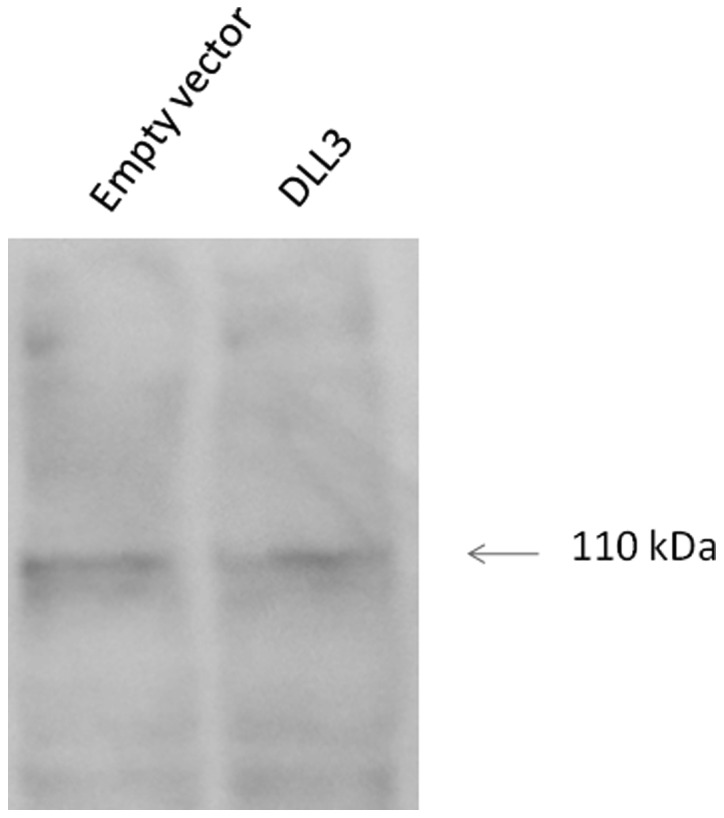
Notch activation by DLL3. Methylation-silenced HuH2 cells were transiently transfected with either DLL3 expression vector or backbone vector and activation of Notch1 was detected by western blot analysis.

Notch1 is a transmembrane protein and the carboxy-terminal Notch1 fragment is released upon binding to a ligand. The resulting activated cytosolic fragment translocates to the nucleus, where it activates transcription. As shown in Fig. 4, western blot analysis showed no significant differences in cleaved Notch1 expression upon overexpression of DLL3.

## Discussion

HCC develops as a result of aberrant genetic and epigenetic events, similarly to other cancers (1). Mutations in tumor suppressor genes, such as *p53*, β-catenin and Axin, are detected in 20–30% of HCC samples (24,25). The epigenetic pathway involves hypomethylation of the HCC genome causing genomic instability, hypermethylation of CpG islands in promoter sequences leading to inactivation of the genes, and histone modification affecting chromatin conformation. In HCC, aberrant promoter hypermethylation associated with gene silencing is observed in genes such as *SOCS-1*, *SOCS-3*, *ASCL*, *p16^INK4a^*, *Ras association domain family 1A* (*RASSF1A*), *placental glutathione S transferase P1* (*GSTP1*) and *E-cadherin*(8–10,26–29).

Yoshikawa *et al*(6) analyzed genomic NotI restriction sites in human HCC and found aberrantly methylated genes, including *SOCS-1*, *SOCS-3*, *ASCL* and *DLL3*, from multiple aberrant NotI sites. *SOCS-1*, known as JAB and SS-1, switches cytokine signaling ‘off’ by means of its direct interaction with Janus kinase (JAK). The authors demonstrated that restoration of *SOCS-1* suppressed growth rate and anchorage-independent growth of cells in which *SOCS-1* is methylation-silenced and JAK2 was constitutively activated (8). *SOCS-3*, which is methylation-silenced in HCC, negatively regulates cell growth and cell migration by enhancing JAK/STAT and FAK signaling (9). The restoration of *ASCL* in methylation-silenced HCC cells induces growth suppression by the induction of apoptosis (10).

In this study, we found that DLL3 is also silenced in HCC cells by aberrant promoter methylation and that the restoration of DLL3 in methylation-silenced HuH2 cells leads to cell growth suppression by induction of apoptosis. We detected apoptosis by the TUNEL method and expression of Annexin V, an early marker for apoptosis. About 5.9% of the DLL3-transfected cells were positive for TUNEL staining, whereas 38.5% of the transfected cells were positive for Annexin V expression. It is possible that methodological differences explain why the ratio of apoptotic cells differed between the two experiments; for detection of Annexin V expression, both adherent and floating cells were subjected to flow cytometry, whereas TUNEL staining was carried out using only adherent cells on the cover slip. In both experiments, apoptotic cells were detected at 3.5 and 2.0-fold higher levels when compared to mock-treated cells. Moreover, the amount of single-stranded DNA was significantly increased in DLL3-transfected cells, and this suggests apoptotic effects for DLL3 in HCC.

DLL3 is a member of the DSL ligands (Delta, Serrate and Lag2), which are type 1 cell surface proteins having multiple tandem epidermal growth factor (EGF) repeats in their extra-cellular domains. In addition, Delta and Serrate proteins contain a conserved cysteine-rich region known as the DSL domain in their extracellular portion. The DSL domain, flanking N-terminal domain, and the first two EGF repeats are required for binding to Notch (30,31). DLL3 is the shortest among the three DLL ligands, with only six EGF-like repeats compared with the eight repeats identified in DLL1 and DLL4. The functions of DLL3 in Notch signaling have been complicated by conflicting reports.

Dunwoodie *et al*(14) reported that injection of *DLL3* RNA into *Xenopus* oocytes is able to inhibit primary neuron formation, as observed in ectopic expression of constitutively active *Notch1*, suggesting that DLL3 is able to activate Notch signaling. However, Ladi *et al* reported that DLL3 does not bind or activate any of the known mammalian Notch receptors when presented in trans, although DLL3 inhibited ligand-induced Notch signaling when coexpressed with Notch at the cell surface in *cis*(15). In addition, Geffers *et al* recently reported that DLL3 does not activate Notch in *D. melanogaster* nor repress DLL1-mediated Notch activation *in vivo*. They also demonstrated that endogenous DLL3 predominantly resides in the Golgi apparatus and is virtually absent from the cell surface, whereas DLL1 is located on the cell surface (16). These results strongly suggest that DLL3 differs functionally from other DSL ligands.

Our western blot analysis demonstrated that overexpression of DLL3 does not increase the expression of cleaved Notch1 in HuH2 cells, thus suggesting that cell growth suppression is induced via a Notch-independent pathway. However, it is not clear how DLL3 induced apoptosis in HCC cells, its Golgi localization may be the key to understanding the novel function of DLL3, including cell growth suppression. In summary, we found that restoration of DLL3 expression induced apoptosis in HuH2 cells via a Notch1-independent pathway.
